# Reduction of aerobic and lactic acid bacteria in dairy desludge using an integrated compressed CO_2_ and ultrasonic process

**DOI:** 10.1007/s13594-015-0241-6

**Published:** 2015-07-10

**Authors:** Tim W. Overton, Tiejun Lu, Narinder Bains, Gary A. Leeke

**Affiliations:** School of Chemical Engineering, University of Birmingham, Edgbaston, Birmingham, B15 2TT UK; Institute of Microbiology & Infection, University of Birmingham, Edgbaston, Birmingham, B15 2TT UK; Sere-Tech Innovation Ltd., Sutton Coldfield, Birmingham, B74 2AD UK

**Keywords:** Desludge, Milk byproduct processing, Sonication, Pressure, Supercritical

## Abstract

Current treatment routes are not suitable to reduce and stabilise bacterial content in some dairy process streams such as separator and bactofuge desludges which currently present a major emission problem faced by dairy producers. In this study, a novel method for the processing of desludge was developed. The new method, elevated pressure sonication (EPS), uses a combination of low frequency ultrasound (20 kHz) and elevated CO_2_ pressure (50 to 100 bar). Process conditions (pressure, sonicator power, processing time) were optimised for batch and continuous EPS processes to reduce viable numbers of aerobic and lactic acid bacteria in bactofuge desludge by ≥3-log fold. Coagulation of proteins present in the desludge also occurred, causing separation of solid (curd) and liquid (whey) fractions. The proposed process offers a 10-fold reduction in energy compared to high temperature short time (HTST) treatment of milk.

## Introduction

There are a number of treatments commonly applied to reduce the viability of bacteria in raw milk to generate products fit for human consumption. Fresh milk is commonly pasteurised using a continuous high temperature short time (HTST) pasteurisation process, typically 72 °C for ≥15 s, or sterilised using an ultra-high temperature treatment (UHT; 150 °C for 1 s). Alternative routes of bacterial deactivation or removal from dairy streams, either industrially or in academic research settings, include centrifugation/bactofugation, reduction in pH (in casein manufacture), sonication, pulsed electric fields and high pressure treatments, each of which have different impacts on the microbiological safety and chemical and sensory properties of the product (Sampedro et al. 2005; Ortega-Rivas and Salmerón-Ochoa 2014). These techniques are typically used in combination to reduce viability of spoilage and pathogenic bacteria to acceptable limits in the final product. Many of the above processes typically require high energy inputs (Pereira and Vicente 2010) or require additional downstream separation processes (for example removal of organic or mineral acids in casein coagulation). Rapid decompression treatment has also been reported in the literature (Foster et al. 1962; Fraser 1951; Hemmingsen and Hemmingsen 1980), but this is only suitable for bacteria that contain gas vacuoles and is therefore not broadly applicable for food processing.

However, many of these treatment routes are not suitable to reduce the bacterial content in some process streams found in the dairy industries. Desludge streams generated by the bactofuge (containing large numbers of bacteria) and separator (containing particles even heavier than the skim, such as sediment, somatic cells and some bacteria) cannot be treated by thermal routes and are currently disposed of at high cost to the processor. A large milk processing site can produce up to 15,000 L · day^−1^ of desludge which is disposed of through water dilution and discharged to sewer at costs of between US$ 150 k and US$ 1.5 M per annum in effluent discharge. In light of this, UK dairy producers have tried local initiatives to treat the desludge; however, several challenges have arisen: bacterially and thermally induced solidification of the desludge, classification of the desludge as waste as it is not effectively pasteurised or heat treated, high off-site waste disposal costs, high transport costs and high tanker cleaning costs.

In this paper, we describe the development of elevated pressure sonication (EPS), a novel technology for the treatment of desludges (Bains and Leeke 2015). The technique uses a combination of elevated CO_2_ pressure (up to 100 bar; lower than high pressure and rapid decompression treatments) and relatively low frequency sonication (20 kHz as opposed to 18 to 500 MHz commonly used in other antibacterial processes). We optimised process parameters (sonicator power, pressure, process time, temperature) to maximise reduction of viable aerobic and lactic acid bacterial counts. The process was optimised in both batch and continuous modes.

## Materials and methods

### Materials

Bactofuge and separator desludges were supplied by a leading dairy company. Samples were collected, stored at 4 °C and used within 24 h of collection; after this time, the bactofuge desludge had phase separated and resultant solids led to blockage of the pump inlet. CO_2_ and N_2_ were obtained from BOC (Wolverhampton, UK) and had given purities of 99.8% *v*/*v* and 99.998%, respectively.

### Batch tests

**Fig. 1:**
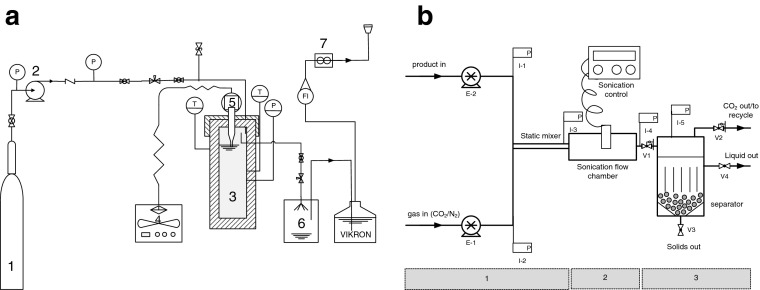
Schematic of experimental setup. **a** Elevated pressure sonication batch tests. *1* gas cylinder, *2* pump, *3* high pressure vessel, *4* ultrasonic controller, *5* ultrasonic probe, *6* collection vessel, *7* mass flow meter. **b** Continuous flow experimental setup. Section 1 = product/gas contact; Section 2 = high pressure sonication chamber; Section 3 = separation

A schematic of the experimental setup used for the high pressure sonication is shown in Fig. 1a. The vessel was a 1-L stainless steel reactor modified for ultrasound (Parr, USA; maximum working conditions 350 °C, 200 bar), with heating jacket, temperature control and thermowell. An ultrasound probe (Sonics and Materials, USA) was integrated into the vessel head and sealed to maintain the pressure at the operating conditions. Although not mechanically agitated, the ultrasound probe allowed sufficient dispersion of the gas with the desludge, as validated by Cenci et al. (2014). Then, 700 mL of desludge was charged into the vessel, which resulted in the probe tip being immersed in the desludge to a depth of 2 mm, and the headspace and ancillaries were evacuated using the gas of choice (CO_2_ or N_2_). The pressure and temperature were then raised to the desired conditions. Sonication was introduced using pulsed mode (1 s on, 1 s off) for the desired time at 20 kHz at different percentages of power (1500 W = 100% power). The internal pressure was observed using a transducer connected to a display (Druck, Leicester, UK) to within ±0.1 bar, while the internal temperature was measured using a type-K thermocouple to within ±0.1 °C. Following the experiment, the solid (curd) and liquid (whey) fractions were collected and analysed for cell viability. For decontamination, the vessel was heated to 125 °C for 30 min and then washed with Virkon (DuPont, Stevenage, UK) to minimise cell contamination between tests.

### Continuous flow apparatus

A similar setup to batch was used for the continuous flow tests. A schematic of the process is shown in Fig. 1b and consists of three sections, (1) product/gas contact, (2) sonication chamber and (3) separator. In (1), the product and gas (CO_2_ or N_2_) were pumped into a stainless steel static mixer where they were contacted. A diaphragm pump (LEWA LDB M9, GmbH) was used for delivering desludge samples into the sonication chamber. Inside the sonication chamber (2) the bacteria present in the product stream were subjected to relatively low frequency sonication (20 kHz). Sonication was provided by a Sonics VCX130 Ultrasonic Processor connected to a 1/4-inch solid Titanium Alloy Probe to give a maximum power output of 130 W. Typical residence times were 30 s to 1 min. The product left the chamber and entered the separation vessel (3) where the solid fraction was separated by gravity from the liquid fraction. The liquid fraction was removed over the weir plates for analysis. The gas disengaged from the liquid and could be vented or recycled. There will be a small amount of gas in the liquid phase which could either be vented (and recycled) or, depending on its use, kept within the liquid. The solid fraction can be periodically removed from the separation vessel using a rotating valve. Desludge is typically produced at the dairy at 50 °C and this temperature was largely used; however, lower temperatures were also evaluated.

### Bacteria viability tests

Bacterial counts were performed on desludge samples before and after processing to generate bacterial deactivation data. Samples were serially diluted in sterile maximum recovery diluent (MRD; 8.5 g · L^−1^ NaCl and 1 g · L^−1^ tryptone in distilled water) and plated onto nutrient agar (Oxoid, Basingstoke, UK; for enumerating total aerobic viable count) or MRS (de Man, Rogosa, Sharpe) agar (Oxoid; for enumerating lactic acid bacteria). Nutrient agar plates were incubated aerobically for 24–48 h at 37 °C and MRS agar plates were incubated in candle jars (microaerobic environment with decreased O_2_ and elevated CO_2_ level) for 24–72 h at 30 °C prior to counting. For analysis of bacterial content of curd samples, fresh wet curd obtained after batch processing was weighed, added to 100 mL of water and stomached using a Seward 400 Circulator Stomacher at 200 rpm for 5 min before serial dilution and plating as above.

### Chemical analysis methods

The Rose Gottlieb method (the standard method of ISO, FAO and WHO for milk fat content measurement) was used to determine the fat content (mg · 100 mL^−1^) in desludge before and after treatment (AOAC 2000; Official method 905.02). Hexane was used to replace petroleum ether.

A MaxSignal® No-Mel Milk Test Kit (Bioo Scientific, Austin, TX) was used to measure protein content in liquid samples before and after treatment. The standard curve was obtained using UV on-line detection using standards provided in the test kit. This kit was not suitable for solid samples, which were sent for analysis at an independent accredited laboratory (Butterworths, London, UK). The classic Kjeldahl method was used in accordance with USP 35,461 Method 1 to determine the nitrogen content in a sample which was then converted into protein content. Liquid samples were also sent to them for comparison with our results.

COD reagent (Merck Millipore Spectroquant) with concentration range of 500–10,000 mg · L^−1^ was used to measure the COD (chemical oxygen demand) of liquid samples before and after treatment.

## Results and discussion

### Batch processing of bactofuge desludge

Power sonication is a technique used to pasteurise many dairy products (Bermúdez-Aguirre and Barbosa-Cánovas 2011); however, it is used at high frequency (typically 18 to 500 MHz) to kill bacteria to acceptable limits. Sonication causes cycles of compression and expansion as well as cavitation in microbial cells. The implosion of bubbles generates points with very high temperatures and pressures which are able to inactivate cells. Sonication is most effective when combined with other microbiological control measures as smaller microbial cells are often more resistant to this treatment (Geciova et al. 2002). One such combination is thermosonication which is undertaken at temperatures between 80 and 95 °C. However, since bactofuge desludge is periodically discharged during dairy processing at 50 °C, this technique would be economically unviable due to the energy input needed to raise the temperature. In addition, processing desludge at high temperatures also results in solidification to a latex-like product which cannot flow and would therefore lead to blockages in an industrial process.

**Fig. 2:**
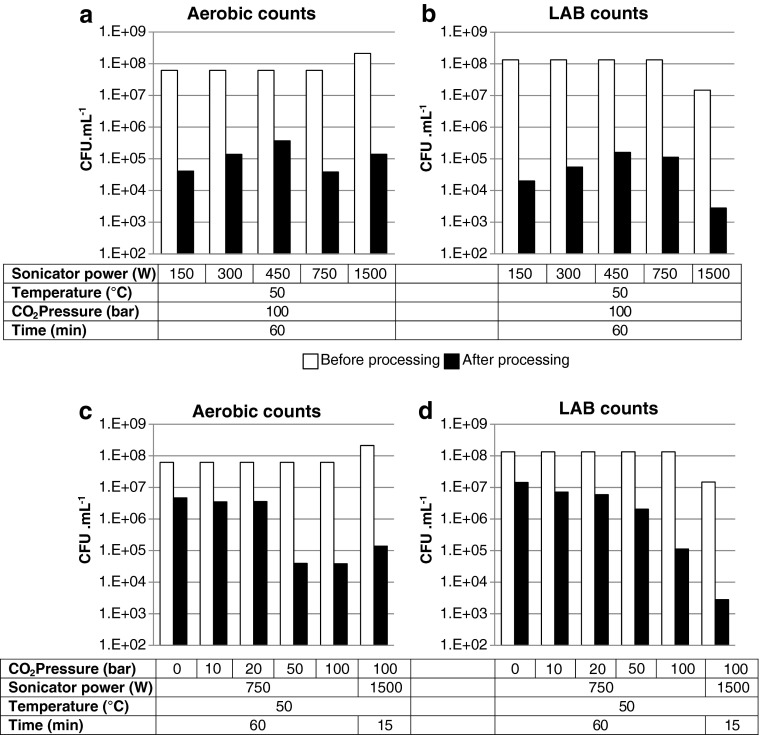
Effect of sonication power (**a**, **b**) and pressure (**c**, **d**) on viable aerobic (**a**, **c**) and lactic acid (**b**, **d**) bacterial counts in bactofuge desludge before and after batch EPS processing. *White bars*, before processing; *black bars*, after processing

**Fig. 3:**
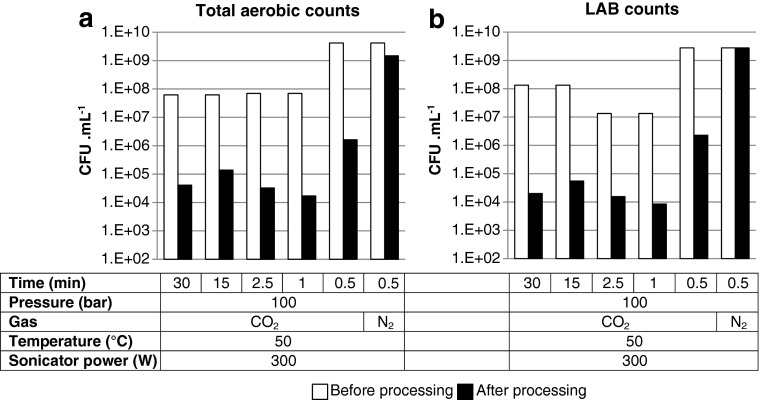
Effect of sonication time and gas on viable aerobic (**a**) and lactic acid (**b**) bacterial counts in bactofuge desludge before and after batch EPS processing. *White bars*, before processing; *black bars*, after processing

In light of the above, lower frequency sonication (20 kHz) was tested in combination with gases at elevated pressures to investigate their effect on the viable cell count of aerobic and lactic acid bacteria in desludge. Lower frequency sonication has lower energy input than the power sonication techniques described above. A series of batch tests were undertaken on bactofuge desludge using the apparatus shown in Fig. 1a and different process conditions (Figs. 2 and 3).

### Effect of sonication power, pressure and processing time on viable cell counts

The sonication power was varied from 1500 to 150 W at a CO_2_ pressure of 100 bar and a processing temperature of 50 °C (Fig. 2a, b). The sonication power could be reduced to 150 W and a 3 to 3.5 log reduction in viable cell count for both aerobic and lactic acid bacteria could still be obtained. Viable cell counts for samples at 300, 450 and 750 W sonication power were undertaken up to 2 and 3 days post-processing. The results demonstrate the effectiveness of the combined treatment to stabilise cell count levels. It is evident that the kill rate of aerobic bacteria is better than that for lactic acid bacteria.

The aerobic and lactic acid bacteria counts were repeated for some tests and were found to have a ST_DEV_ of 11% for aerobic bacteria and a ST_DEV_ of 21% for lactic acid bacteria. The reproducibility of aerobic bacteria is better than for lactic acid bacteria although both are within an acceptable experimental error.

In light of the results shown in Fig. 2a and b, tests were undertaken at lower pressure in order to reduce the pressure design requirements for the treatment process. Figure 2c and d shows that a pressure ≥50 bar was required to achieve a 3.5-log-fold reduction in aerobic viable cell count levels, whereas a pressure ≥100 bar was required to achieve a 3.5-log-fold reduction in lactic acid viable cell count levels. In the absence of any gas pressure (0 bar, ambient pressure conditions), sonication had a small effect on viable cell count with approximately 1-log-fold reduction in both total aerobic and LAB counts. The combination of CO_2_ and sonication is therefore necessary for the process to be effective.

The effect of sonication time was investigated in order to reduce the energy requirement and processing time of the treatment process. Figure 3 shows that the time could be reduced to 1 min with 3-log-fold reductions in viable cell counts still being achieved. The sonication time was further reduced to 0.5 min (with all other conditions the same) and showed favourable results toward cell death (∼3.4-log-fold reduction in aerobic counts, ∼3.1-log-fold reduction in LABs). This short process time makes the EPS treatment process potentially suitable for continuous processing of desludge. Tests were also undertaken in the presence of N_2_ at 100 bar instead of CO_2_. The results show that N_2_ had an insignificant effect on cell viability, demonstrating that CO_2_ was required for process effectiveness.

### Characteristics of processed desludge

**Fig. 4:**
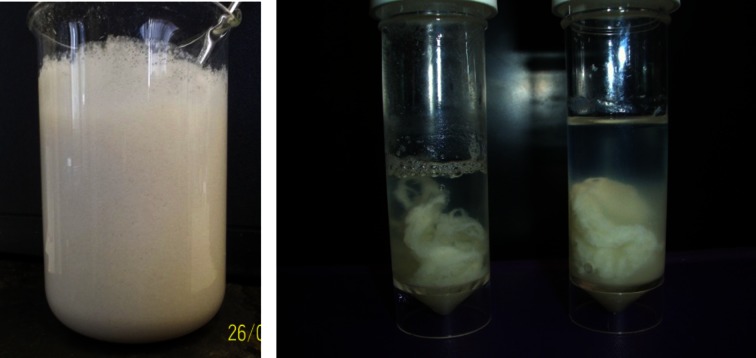
Images of bactofuge desludge pre- (*left*) and post- (*right*) EPS treatment. Process conditions: batch; 100 bar CO_2_, 300 W pulsed sonication power, 3 min

Photographs of untreated and treated bactofuge desludge are shown in Fig. 4. Due to the relatively low temperature, frequency of the sonication and power input, the solid fraction did not form a latex-like material following processing and exhibited the ability to flow due to the presence of the liquid phase. During exposure to high pressure CO_2_, the pH in the desludge would have been reduced to approximately 3 at the process conditions (Leeke et al. 2005). The literature reports that when sonication is combined with a low pH environment, organisms are not able to grow (Bermúdez-Aguirre and Barbosa-Cánovas 2011). It is thought that the combination of sonication, pressure and lowering of pH results in bacterial death in this process, in a similar manner to the hurdle technology approach used in food processing. The lowered pH also caused the desludge to separate into liquid (whey) and solid (curd) fractions, without the addition of solid chemicals dissolved in the process liquor. The lack of bacterial killing in the presence of N_2_ (Fig. 3) may be explained by its lower solubility in water (assumed to be the main liquid component); 1.011 cm^3^ STP · g^--1 ^water as opposed to 25.6 cm^3^ STP · g^--1^ water for CO_2_ at 100 bar and 50 °C (Wiebe and Gaddy 1939; Wiebe and Gaddy 1940; Wiebe et al. 1933). In addition, the chemical properties of N_2_ would not lead to a decrease in pH during processing.

Prior to treatment the COD of the desludge was 1.2 million mg · L^−1^ making it costly to dispose of. After high pressure sonication treatment, the COD of the whey fraction was 33,000–42,000 mg · L^−1^ with the remainder in the solid fraction. Thus, the treatment does not lower the COD but partitions it into the solid fraction. This offers an added advantageous separation process and retains the majority of the COD in a dewatered solid fraction making it suitable for use as a value-added product such as animal feed or an energy source. The whey fraction is also likely to contain valuable components, similar to those found in cheese whey (de Wit 2001).

### Energy requirements for elevated pressure sonication treatment

Table 1 shows the energy requirements of the sonicator (kJ · L^−1^) to treat 700 mL of bactofuge desludge using the batch EPS process. Selecting test 6 as the optimal case, the additional energy requirements for CO_2_ compression and desludge compression were calculated as 6.1 and 8.6 kJ · L^−1^, respectively, giving the total energy required to process 1 L of desludge as 24.7 kJ. A comparison is made with the energy required to pasteurise milk using HTST treatment. The EPS process requires less energy than that required to pasteurise milk and is therefore highly attractive as a commercial process.

**Table 1:** Energy requirement to treat bactofuge desludge at different conditions

Method/test number		Pressure (bar)	Temperature (°C)	Power (W)	Time (min)	Energy needed (kJ · L^−1^)
EPS	Test 1	100	50	1500	15	854
	Test 2	50	50	750	30	701
	Test 3	100	50	750	30	1241
	Test 4	100	50	300	15	171
	Test 5	100	50	300	2.5	34
	Test 6	100	50	300	1	10
Pasteurisation of milk by HTST		Continuous	72		0.25	196
		Batch	63		15	158
		High fat	75		0.25	209

All tests gave a 3- to 3.5-log-fold reduction in total aerobic counts. All tests, except Test 2, gave a 3- to 3.5-log-fold reduction in lactic acid bacteria. Energy needed for EPS process based on sonicator energy input only. HTST based on process without heat integration

*HTST* high temperature short time

### Continuous tests on desludge samples

**Fig. 5:**
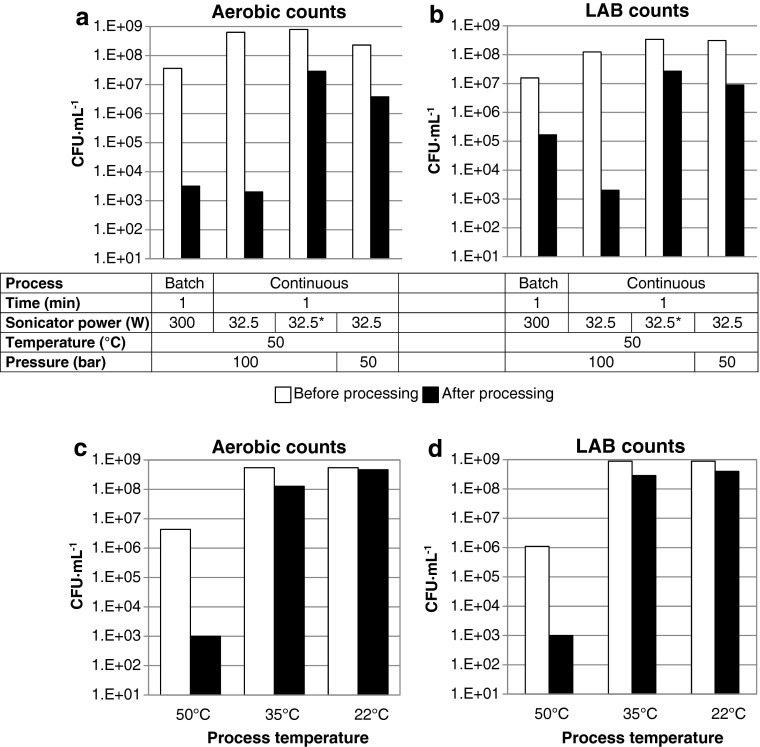
**a**, **b** The effect of different batch and continuous EPS processing conditions on aerobic (**a**) and lactic acid (**b**) bacterial counts. *White bars*, before processing; *black bars*, after processing. Batch process was performed on bactofuge desludge (Test 6 in Table 1), continuous process was performed on a 1:1 mixture of bactofuge and separator desludges. Continuous process used flow rates of 10 mL · min^−1^ desludge and ∼4 g · min^−1^ CO_2_, giving a residence time of 1 min. *Pulsed sonication, 1 s on and 1 s off. **c**, **d** Effect of processing temperature on aerobic (**c**) and lactic acid (**d**) bacterial counts. Process conditions were as follows: continuous process; pressure, 100 bar; temperature, as shown; sonicator power = 32.5 W; sample flow, 10 mL · min^−1^, CO_2_ flow, 4 g · min^--1^

Using the optimum batch conditions identified above, the process was modified to treat desludge under continuous flow conditions (Fig. 1b) to test its suitability for large volume processing. Tests were carried out on both bactofuge and separator desludge. Since dairy plants produce similar quantities of separator and bactofuge desludge, a mixture containing 50:50 vol.% of both desludges was tested using the continuous EPS process (Fig. 5).

Similar to the batch tests (Figs. 2 and 3), lowering the pressure or sonicator energy input resulted in poorer reductions in viable cell numbers. Cell viability was significantly reduced for the separator sample with the flow process giving an even better reduction in viable counts than batch processing, possibly from the enhanced mixing induced in the flow process. In addition, pulsed sonication was also tested. The flow rate of the samples was kept constant at 10 mL · min^−1^ while the sonication was either constant (18 kJ · L^−1^ energy input) or pulsed modality (11.9 kJ · L^−1^ energy input). Temperature (50 °C), pressure (100 bar) and CO_2_ flow rate (∼4 g · min^−1^) were the same for both tests. For either continuous or pulsed sonication, aerobic bacterial counts reduced from 4.3 × 10^6^ CFU · mL^−1^ before treatment to <10^3^ CFU · mL^−1^ after treatment; LAB counts similarly reduced from 1.1 × 10^6^ CFU · mL^−1^ before treatment to <10^3^ CFU · mL^−1^ after treatment (see Fig 5c and d).

The effect of temperature on continuous EPS processing of bactofuge desludge was tested. Tests were carried out at 50 and 35 °C (conditions where CO_2_ was supercritical) and 22 °C (where CO_2_ was liquid). It was discovered that temperature and state play a key role in desludge treatment (Fig. 5c, d). Both liquid CO_2_ and supercritical CO_2_ close to the critical point (31 °C) did not effectively kill bacteria.

### Shelf-life of treated and untreated bactofuge desludge

**Fig. 6:**
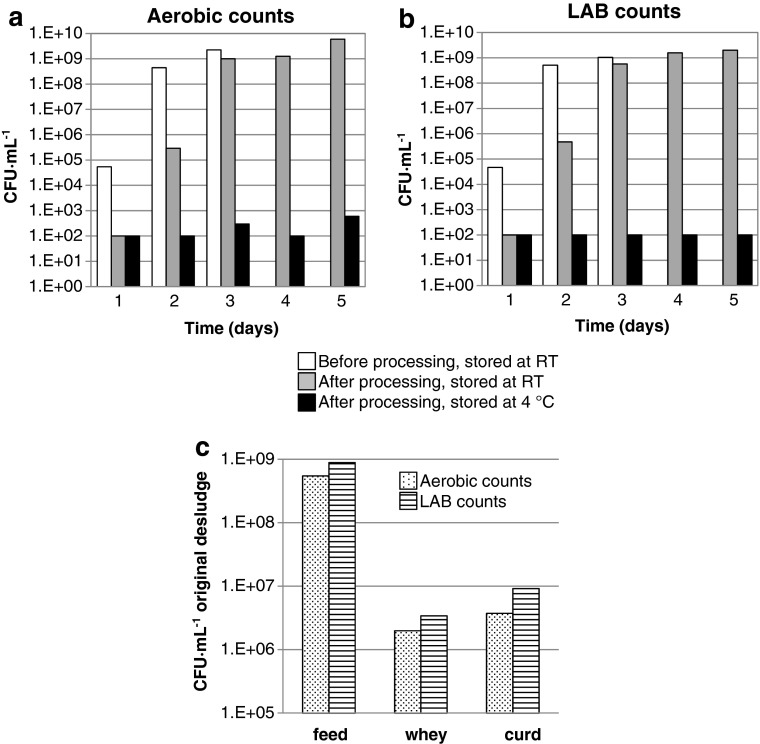
**a**, **b** Shelf-life of bactofuge desludge before (feed) and after processing. Total aerobic bacteria (**a**) and lactic acid bacteria (**b**) counts were determined for bactofuge desludge without EPS treatment (*white bars*) and with EPS treatment, after storage at room temperature (*grey bars*) or 4 °C (*black bars*) for up to 5 days. Processing conditions: 100 bar CO_2_; 50 °C; 32.5 W sonicator power; (pulsed) 1 min residence time; energy input of 12 kJ · L^−1^. **c** Partitioning of bacteria into curd and whey fractions following EPS processing. Bactofuge desludge was processed (batch EPS process; 100 bar CO_2_; 50 °C; 300 W sonicator power pulsed 1 s on, 1 s off; 2 min; energy input of 19 kJ · L^−1^) and viable aerobic (*spotted bars*) and LAB (*striped bars*) determined before processing and after processing in the whey and curd (following stomaching) fractions. Numbers of bacteria are expressed as CFU per millilitre of original desludge sample, taking into consideration the quantity of curd and whey fractions generated during EPS processing

The shelf-life of untreated bactofuge desludge was compared to treated desludge (100 bar CO_2_, 50 °C, 32.5 W power, pulsed, 1 min residence time and energy input of 12 kJ · L^−1^) in terms of lactic acid and aerobic counts. Treated and untreated samples were incubated at either room temperature or 4 °C and viable counts determined at daily intervals (Fig. 6a, b). EPS-treated bactofuge desludge could be stored at 4 °C for 5 days without an obvious increase in LAB counts and with less than 1 log increase in total aerobic counts. The treated sample stored at room temperature attained similar viable counts to the untreated sample after 1-day storage at room temperature. After 3 days of storage at room temperature, both untreated and treated bacterial counts were high; the untreated desludge solidified, after which it could not be further assessed.

### Analysis of curd and whey fractions

The volume of curd and whey fractions formed after batch processing of bactofuge desludge (100 bar, 50 °C, 300 W power, 1:1 pulse for 1 min) were measured. Typically the liquid fraction was between 71 and 77% with a 22–29% wet solid fraction. The percentage mass of the dry solid was found to be approximately 5 to 8% and was determined by drying the wet solid sample in an oven at 25 °C for 15 consecutive days until there was no mass change. The drying process caused gradual hardening of the sample and resulted in a colour change from white cream to semi-transparent. The solid merged together when drying to form a hard, strong and hollow material of roughly the shape of its container.

The bacterial content of the curd and whey fractions was determined; the curd fraction was stomached to release bacteria (Fig. 6c). Both aerobes and LABs were preferentially partitioned into the curd fraction.

### Fat and protein content of desludge and processed fractions

The fat content of the curd and whey fractions obtained after batch processing was determined using the Rose Gottlieb method (Table 2) and compared to values found in the literature (de Wit 2001). Dry solids were obtained after drying at 25 or 70 °C. Protein concentrations of EPS-derived whey fractions determined using the Rose Gottlieb method were comparable to the protein content of casein, lactic acid and cheese whey (de Wit 2001). Protein concentrations of whey samples were also determined by an external laboratory; data were comparable between the two analysis methods. The protein content of cheese is typically between 17 and 42% with the majority of cheeses being between 20 and 32%. The fresh wet curd obtained from the EPS process contained 35.3% protein and so has potential for use as animal feed or other purposes.

**Table 2:** Fat and protein content of desludge samples before and after batch EPS treatment

Sample	State	Fat content mg · 100 mL^−1^ (liquid) or mg · 100 g^−1^ (solid)		Protein content %		
		This work	Literature value	This work	External lab	Literature value
Unprocessed bactofuge desludge	Liquid	60	–	6.89	10.55	–
Whey (sample 1)	Liquid	19.6	30–50	0.67	1.1	0.6–0.62^a^
Whey (sample 2)	Liquid	16.6	30–50	0.53	0.7	0.6–0.62^a^
Fresh curd of sample 2	Wet solid	455.2	ND	33.7	35.3	ND
Curd of sample 1 dried at 25 °C	Solid	ND	ND	ND	77.5	ND
Curd dried at 70 °C	Solid	ND	ND	ND	70.95	ND
Unprocessed separator desludge	Liquid	143.6	ND	ND	ND	ND
Milk^b^	Liquid	ND	4110	ND	ND	3.3

All tests processed bactofuge desludge in batch EPS process at 100 bar, 50 °C, 300 W power for 1 min; samples 1 and 2 are repeat analyses to show reproducibility. Fat content units depend on solid or liquid samples. Fat content compare our data (Rose Gottlieb method) and literature data (de Wit 2001); protein content compare our data (MaxSignal® No-Mel Milk Test Kit), external laboratory data (Kjeldahl method) and literature data (de Wit 2001)

*ND* not determined

^a^Range of values for casein, lactic acid and cheese whey

^b^Data for milk from literature (de Wit 2001)

The protein content of the bactofuge curds were corrected on a dry basis and are shown in Table 3. The calculation was based on a 60% moisture content in the fresh curd, 12% moisture content in the 25 °C dried curd and 8% moisture content in the 70 °C dried curd. The data suggest that drying the processed curd at 70 °C might damage the protein in the curd. Yellow-brownish areas were seen on the 70 °C dried curd surface and suggest that the Maillard reaction had occurred in the presence of the water leading to protein damage; its lower protein content agreed with this assumption.

**Table 3:** Protein determination of bactofuge curd on dry weight basis

Sample	Protein %	Protein % (dry base)
Fresh (wet) curd	35.3	88
25 °C dried curd	77.5	88
70 °C dried curd	70.95	77

## Conclusion

Elevated pressure sonication (EPS) offers a way to effectively process bactofuge desludge. Pressure alone or sonication alone do not kill bacteria; however, in combination, a 3-log-fold decrease in aerobic bacteria and 3.5-log-fold decrease in lactic acid bacteria can be obtained. Low CO_2_ pressure (50 bar) can be used to reduce viable aerobic bacteria whereas high CO_2_ pressure (100 bar) must be used to reduce numbers of lactic acid bacteria. High pressure sonication in the presence of N_2_ resulted in insignificant cell death. Sonication power as low as 150 or 300 W can be used to kill aerobic and lactic acid bacteria in a very short treatment time (<0.5 min). In comparison to HTST of fresh milk, the technology leads to a 10-fold reduction in energy requirement. Comparison with other emerging dairy processing techniques such as high pressure or pulsed electric field treatment is difficult as they have not been applied to desludge treatment; this could form the basis of future work.

The good flow properties of the desludge post-processing meant that this treatment technique has real potential for industries that rely on high volume processing. The treatment of desludge by conventional thermal treatments leads to a latex-like product that cannot flow. The curd fraction could potentially be used as a food or feed product (e.g. cheese or animal feed), or could be used to raise energy by digestion. The whey fraction has value in its own right as a potential source of bioactive peptides. The induced separation can therefore also be seen as a dewatering process of the solid curd fraction. The EPS process thereby generates a value product and the technique has potential to cross over to other dairy products, brewery waste and beverage processing. If the product is considered as a food product, further testing (e.g. for endotoxins in the lysate) and approval is required.
